# Effects of red and blue light on leaf anatomy, CO_2_ assimilation and the photosynthetic electron transport capacity of sweet pepper (*Capsicum annuum* L.) seedlings

**DOI:** 10.1186/s12870-020-02523-z

**Published:** 2020-07-06

**Authors:** Yan Li, Guofeng Xin, Chang Liu, Qinghua Shi, Fengjuan Yang, Min Wei

**Affiliations:** 1 College of Horticultural Science and Engineering, Shandong Agricultural University, Tai’an, China; 2 Scientific Observing and Experimental Station of Environment Controlled Agricultural Engineering in Huang-Huai-Hai Region, Ministry of Agriculture, Tai’an, China; 3 Shandong Collaborative Innovation Center of Fruit & Vegetable Quality and Efficient Production, Tai’an, China; 4 State Key Laboratory of Crop Biology, Tai’an, 271018 China; 5 Entomology and Nematology Department, University of Florida, 1881 Natural Area Dr, Gainesville, FL USA

**Keywords:** Sweet pepper (*Capsicum annuum* L.), Light quality, Anatomy, Photosynthesis, CO_2_ assimilation

## Abstract

**Background:**

The red (R) and blue (B) light wavelengths are known to influence many plant physiological processes during growth and development, particularly photosynthesis. To understand how R and B light influences plant photomorphogenesis and photosynthesis, we investigated changes in leaf anatomy, chlorophyll fluorescence and photosynthetic parameters, and ribulose-1, 5-bisphosphate carboxylase/oxygenase (Rubisco) and Calvin cycle-related enzymes expression and their activities in sweet pepper (*Capsicum annuum* L.) seedlings exposed to four light qualities: monochromatic white (W, control), R, B and mixed R and B (RB) light with the same photosynthetic photon flux density (PPFD) of 300 μmol/m^2^·s.

**Results:**

The results revealed that seedlings grown under R light had lower biomass accumulation, CO_2_ assimilation and photosystem II (PSII) electron transportation compared to plants grown under other treatments. These changes are probably due to inactivation of the photosystem (PS). Biomass accumulation and CO_2_ assimilation were significantly enriched in B- and RB-grown plants, especially the latter treatment. Their leaves were also thicker, and photosynthetic electron transport capacity, as well as the photosynthetic rate were enhanced. The up-regulation of the expression and activities of Rubisco, fructose-1, 6-bisphosphatase (FBPase) and glyceraldehyde-phosphate dehydrogenase (GAPDH), which involved in the Calvin cycle and are probably the main enzymatic factors contributing to RuBP (ribulose-1, 5-bisphosphate) synthesis, were also increased.

**Conclusions:**

Mixed R and B light altered plant photomorphogenesis and photosynthesis, mainly through its effects on leaf anatomy, photosynthetic electron transportation and the expression and activities of key Calvin cycle enzymes.

## Background

Light is one of the most important environmental factors affecting plant growth and development [1]. Using light rather than chemicals to control plant architecture can reduce the environmental impacts [2]. Light affects the photosynthetic characteristics of seedlings by regulating chloroplast and anatomy development, and through its influence on key enzyme activities and the related expression of genes involved in the Calvin cycle, etc. [3–6].

Photosynthesis is the green engine that powers life on Earth, as it is the only biological process that allows plants, etc., to convert light energy into chemical energy [7]. Improving photosynthesis is critical to maintaining sufficient dry biomass accumulation. It is well known that in addition to light intensity and photoperiod, light quality, namely, light color or wavelength, exerts a significant effect on regulating plant growth and photosynthesis [8–12]. Specific light qualities have precise effects on plants. For example, blue (B) and red (R) light are the most effectively utilized wavelengths during plant photosynthesis because the absorption spectra of the photosynthetic pigments mainly focus on the B (400–500 nm) and R (600–700 nm) light spectra. Therefore, their utility and regulatory mechanisms have always been important areas of research [13, 14].

A few studies have used R and B light to examine the effects of light quality on anatomy, photosynthesis and morphology of plants. In general, R light plays an important role in controlling the functions of the chloroplast, stem and petiole growth and the reproductive system [15, 16]. B light affects plant growth, leaf expansion, photomorphogenesis, stomatal opening, photosynthesis and pigment accumulation [17, 18]. Furthermore, it is shown that plants grown under B light have higher stomatal conductance, lager chlorophyll (Chl) *a*/*b*, greater photosystem (PS) activity and photosynthetic electron transport ability, higher levels of ribose-1, 5-bisphosphate carboxylase/oxygenase (Rubisco) activity and expression of genes related to Calvin cycle than those plants grown under R light [19, 20].

The Calvin cycle which occurs during the process of photosynthesis consists of light-independent redox reactions which happens in the stroma of chloroplasts and exerts a key effect on photosynthetic carbon fixation. The efficiency of carbon assimilation is affected by the regeneration rate of ribulose-1, 5-bisphosphate (RuBP). Rubisco is a key enzyme in plant photosynthesis that controls both carbon dioxide and carbon fixation [21]. This set of reactions is catalyzed by Rubisco as well as other corresponding key enzymes and finally converts carbon dioxide and water into organic sugars. According to the previous researches, light quality exerts an impact on photosynthetic property by regulating the expression of these genes related [22, 23].

It has also been shown that monochromatic R or B light does not satisfy normal plant growth requirements and the absence of one of the two light qualities creates photosynthetic inefficiencies [24]. Various studies have found that mixed R and B light is an effective lighting source that improves plant development and a suitable proportion of R and B light accelerates photosynthesis and the growth of tomato, cucumber and sweet pepper, etc. [24–26]. Leaf anatomy may directly influence light capture by its leaf thickness as well as by the differentiation of palisade and spongy mesophyll. Earlier report showed that leaf thickness increased when R light was supplemented with B light [27]. Furthermore, Klein [28] and Naznin [26] found that mixed R and B light led to higher Chl *a*, *b* and total Chl levels, an improved electron transport rate (ETR) and an early onset of non-photochemical quenching (NPQ), all of which lead to increases in photosynthetic efficiency. Therefore, mixed R and B light is now used in research studies and commercial horticulture because of their effective photosynthetic wavelengths at the leaf level [29, 30]. Despite these achievements, the specific photosynthesis processes in plants affected by mixed R and B light remains largely unknown.

The popularity of sweet pepper (*Capsicum annuum* L.) for fresh market consumption or in ready-to-eat food has risen significantly during the past decades and these peppers are mostly produced in protected environments [31]. Mixed R and B light has an apparent influence on the growth and physiology of pepper plants [26, 32, 33]. Gaining a more complete mechanistic picture of how plants adapt and respond to R and B light quality is important since light quality plays important roles in growth and physiology. In addition, a better understanding of the leaf anatomy, CO_2_ assimilation and photosynthetic electron transport that influence responses to R and B light can improve the photosynthetic efficiency and assist in developing better methods to evaluate plant responses to light quality. Recently, light-emitting diodes (LEDs), which are light sources that have a high photosynthetic efficiency, have been successfully used in scientific research and protected horticulture [34–36]. Our previous studies have found that a suitable proportion of mixed R and B light (light intensity of R:B = 3:1, RB) accelerated pepper seedlings’ photosynthesis and growth. The objective of this study was to examine how R and/or B light sources affected pepper seedling photomorphogenesis, photosynthetic characteristics, as well as the transcriptional and translation levels of key enzymes in the Calvin cycle.

## Results

### Plant morphology and biomass accumulation under different light treatments

**Fig. 1:**
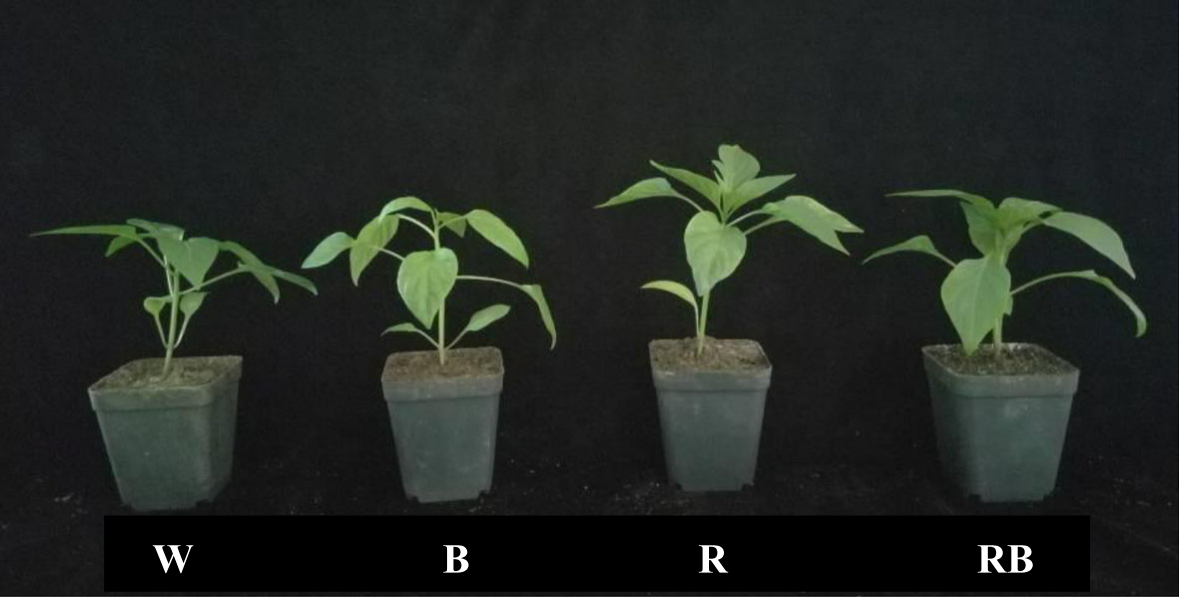
Effects of different light treatments on plant morphology of sweet pepper seedlings at 28 day after treatment. W, white light; R, monochromatic R light; B, monochromatic B light; RB, mixed R and B light of 3:1

**Fig. 2:**
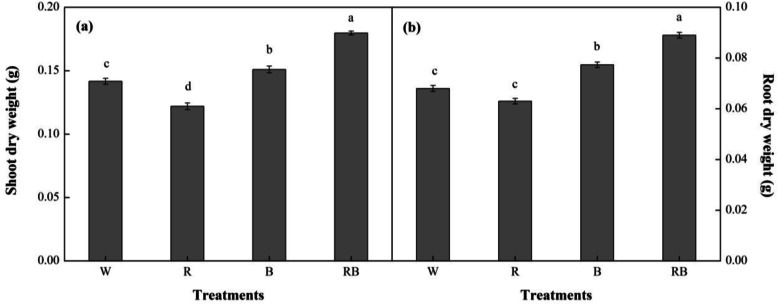
Effects of different light treatments on (**a**) shoot dry weight and (**b**) root dry weight of sweet pepper seedlings at 28 day after treatment. Data are presented as means ± SE, *n* = 3. Different letters indicate significant differences between values (*p* < 0.05). W, white light; R, monochromatic R light; B, monochromatic B light; RB, mixed R and B light of 3:1

A visual overview of the influence of monochromic and mixed R and B light on morphology of sweet pepper seedlings at 28 day (d) after treatment (DAT) was shown in Fig. 1 and Supplementary Fig. 2 and the differences among different treatments were significant. The plant shoot dry weight (DW) under RB was significantly increased compared with W (*P* < 0.05), and it was also higher than that under other treatments, whereas, R light produced the lowest DWs (Fig. 2a). The root DWs showed similar trends under all the treatments (Fig. 2b).

### Leaf anatomy under different light treatments

**Fig. 3:**
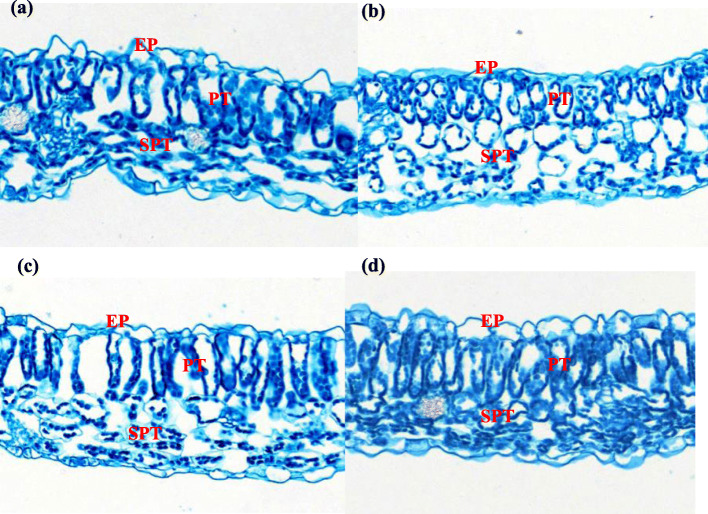
Effects of different light treatments: (**a**) white light; (**b**) monochromatic R light; (**c**) monochromatic B light; (**d**) mixed R and B light of 3:1 on leaf sectioning anatomy of sweet pepper seedlings at 28 day after treatment. Images of leaf sectioning anatomy are at the same magnification. The images were taken at 200 × magnification. EP, epidermis cell; PT, palisade mesophyll tissue; SPT, spongy mesophyll tissue

Table 1 and Fig. 3 showed that R and B light had a significant effect on the anatomical structure of pepper leaves. Leaf thickness was the highest under RB, followed by B and W, while the thinnest leaves were found under R light. Furthermore, compared to W, the thickness of palisade mesophyll tissue (PT), spongy mesophyll tissue (SPT) and the upper epidermis were significantly greater under RB treatment (*P* < 0.05). These three parameters increased by 26, 19 and 22%, respectively, but they were significantly reduced by R light. Thinner lower epidermal thicknesses were found under R, whereas the epidermis tended to be thicker under RB although they were not significantly different from W. The effect on the PT and SPT ratio was not strong (*P* > 0.05) and the thinnest cell layers occurred under R.

**Table 1:** Effects of different light treatments on leaf anatomy of sweet pepper seedlings at 28 day after treatment

Treatments	Leaf thickness (μm)	Palisade mesophyll issue thickness (μm)	Spongy mesophyll issue thickness (μm)	Upper epidermis thickness (μm)	Lower epidermis thickness (μm)	Palisade mesophyll tissue/spongy mesophyll tissue ratio
W	122.54 ± 4.92 b	39.73 ± 2.11 b	67.92 ± 3.02 b	8.35 ± 0.39 b	6.21 ± 0.11 ab	0.59 ± 0.06 ab
R	103.25 ± 3.78 c	30.21 ± 1.32 c	59.03 ± 2.82 c	6.23 ± 0.15 c	5.88 ± 0.19 b	0.51 ± 0.03 b
B	130.22 ± 3.15 b	43.33 ± 1.87 b	73.24 ± 1.45 b	7.96 ± 0.27 b	6.07 ± 0.14 b	0.59 ± 0.02 a
RB	146.90 ± 5.21 a	50.07 ± 2.56 a	81.02 ± 2.56 a	10.18 ± 0.11 a	6.42 ± 0.12 a	0.62 ± 0.04 a

Data are presented as means ± SE, *n* = 3. Different letters indicate significant differences between values (*p* < 0.05). *W* white light, *R* monochromatic R light, *B* monochromatic B light, *RB* mixed R and B light of 3:1. The same as below

### Photosynthetic light- and CO_2_-response curves under different light treatments

**Fig. 4:**
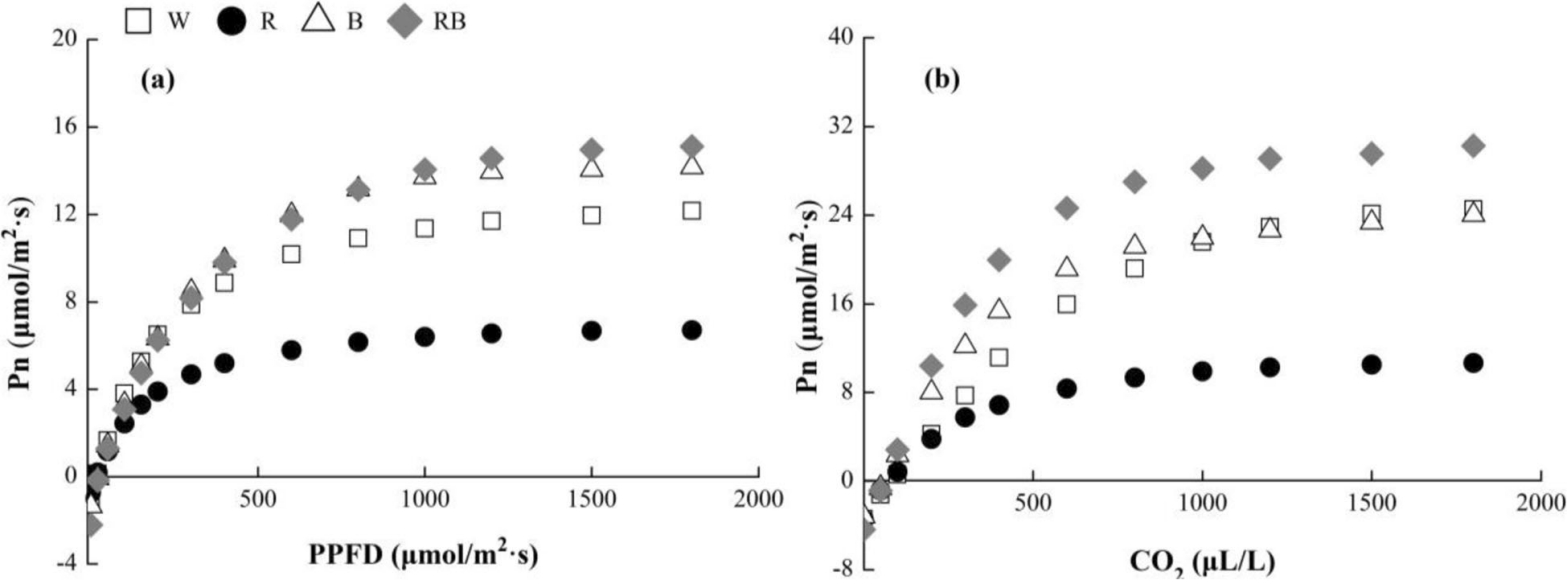
Effects of different light treatments on (**a**) photosynthetic light- and (**b**) CO_2_-response curves of sweet pepper seedlings at 28 day after treatment. Pn, net photosynthetic rate; PPFD, photosynthetic photon flux density; W, white light; R, monochromatic R light; B, monochromatic B light; RB, mixed R and B light of 3:1. □ W; ● R; △ B; ◆ RB

Both of the net photosynthetic rate (Pn) of the leaves increased rapidly along with the increment in PPFD (Fig. 4a) and CO_2_ concentration (Fig. 4b) at the initial stage, after that, their increasing tendency gradually became stable. The highest Pn-PPFD response curve value was detected under RB, followed by B and W, whereas R produced the lowest value. Furthermore, different light treatments produced similar trends for Pn-CO_2_. The apparent quantum efficiency (AQY), light saturation point (LSP), light-saturated maximum (Pn_max_), carboxylation efficiency (CE) and CO_2_ saturation point (CSP) levels and the maximum RuBP regeneration rate were significantly higher under RB (*P* < 0.05) than those under W, whereas, the light compensation point (LCP) and CO_2_ compensation point (CCP) values were decreased under this treatment (Table 2 and Table 3).

**Table 2:** Effects of different light treatments on photosynthetic light-response curve parameters of sweet pepper seedlings at 28 day after treatment

Treatments	AQY (μmol/m^2^·s)	LCP (μmol/m^2^·s)	LSP (μmol/m^2^·s)	Pn_max_ (μmol/m^2^·s)
W	0.051 ± 0.003 b	26.6 ± 2.36 a	729 ± 38.42 c	13.0 ± 0.23 b
R	0.030 ± 0.002 c	27.3 ± 2.11 a	520 ± 29.14 d	6.1 ± 0.45 c
B	0.050 ± 0.002 b	23.7 ± 1.82 b	924 ± 27.68 b	15.2 ± 0.62 a
RB	0.056 ± 0.001 a	22.8 ± 2.91 b	968 ± 28.35 a	16.3 ± 0.67 a

*AQY* apparent quantum efficiency, *LCP* light compensation point, *LSP* light saturation point, *Pn*_*max*_ light-saturated maximum

**Table 3:** Effects of different light treatments on photosynthetic CO_2_-response curve parameters of sweet pepper seedlings at 28 day after treatment

Treatments	CE (mol/m^2^·s)	CCP (μmol/m^2^·s)	CSP (μmol/m^2^·s)	Maximum RuBP regeneration rate (μmol/m^2^·s)
W	0.047 ± 0.006 b	81 ± 5.69 b	1087 ± 25.38 c	23.1 ± 3.46 b
R	0.032 ± 0.004 c	92 ± 3.21 a	1213 ± 12.34 b	11.0 ± 1.14 c
B	0.057 ± 0.009 b	57 ± 3.00 c	1040 ± 17.56 d	21.4 ± 1.96 b
RB	0.066 ± 0.003 a	61 ± 6.66 c	1443 ± 21.39 a	39.5 ± 1.06 a

*CE* carboxylation efficiency, *CCP* CO_2_ compensation point, *CSP* CO_2_ saturation point

### Chlorophyll a fluorescence and the chlorophyll fluorescence transients under different light treatments

**Fig. 5:**
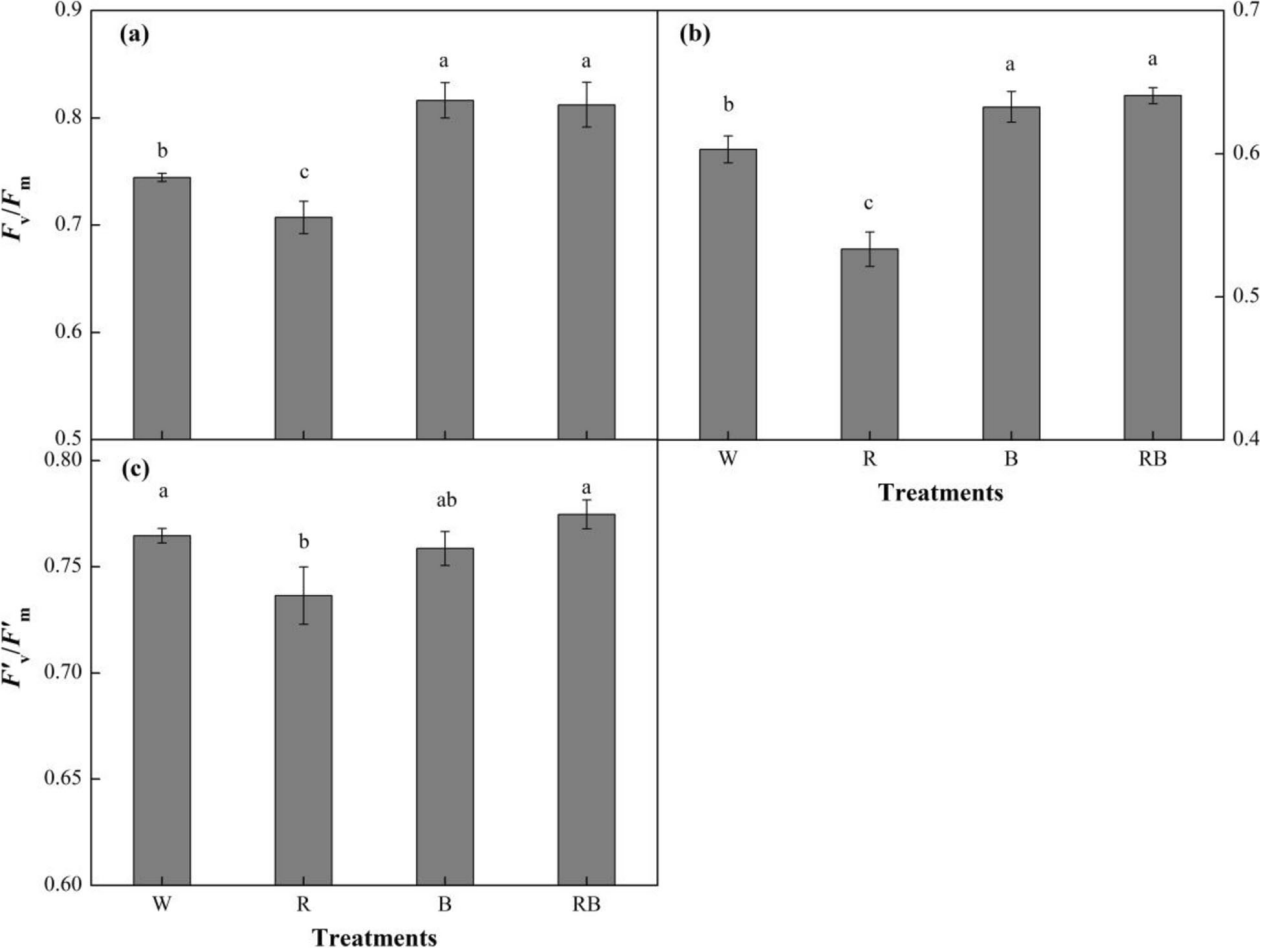
Effects of different light treatments on chlorophyll fluorescence parameters: (**a**) *F*_v_/*F*_m_, maximum photochemical efficiency of PSII; (**b**) *Φ*_PSII_, actual PSII photochemical efficiency; (**c**) *F*’_v_/*F*’_m_, maximum photochemical efficiency of PSII under light adaptation of sweet pepper seedlings at 28 day after treatment. Data are presented as means ± SE, n = 3. Different letters indicate significant differences between values (*p* < 0.05). W, white light; R, monochromatic R light; B, monochromatic B light; RB, mixed R and B light of 3:1

The effects of R and B light on the pepper seedling Chl fluorescence parameters were shown in Fig. 5. *F*_v_/*F*_m_, which represents the greatest light conversion efficiency or the maximum quantum yield of PS II, was significantly higher under RB and B than that under W and there were no significant differences between RB and B treatments (Fig. 5a). Furthermore, this parameter significantly declined under R (*P* < 0.05). *Φ*_PSII_ represents the actual conversion efficiency of PS II or the actual quantum yield and it showed a similar reaction to the four light quality treatments (Fig. 5b). *F*’_v_/*F*’_m_ indicates how efficiency the excitation energy is captured by open photosystem II (PSII) reaction centers and it was enhanced in RB-grown seedlings, followed by W and B, and there were no significant differences among these three treatments (*P* > 0.05) (Fig. 5c). However, seedlings grown under R light had significantly lower *F*’_v_/*F*’_m_ values (*P* < 0.05), and no significant difference was found between R and B treatments.

**Fig. 6:**
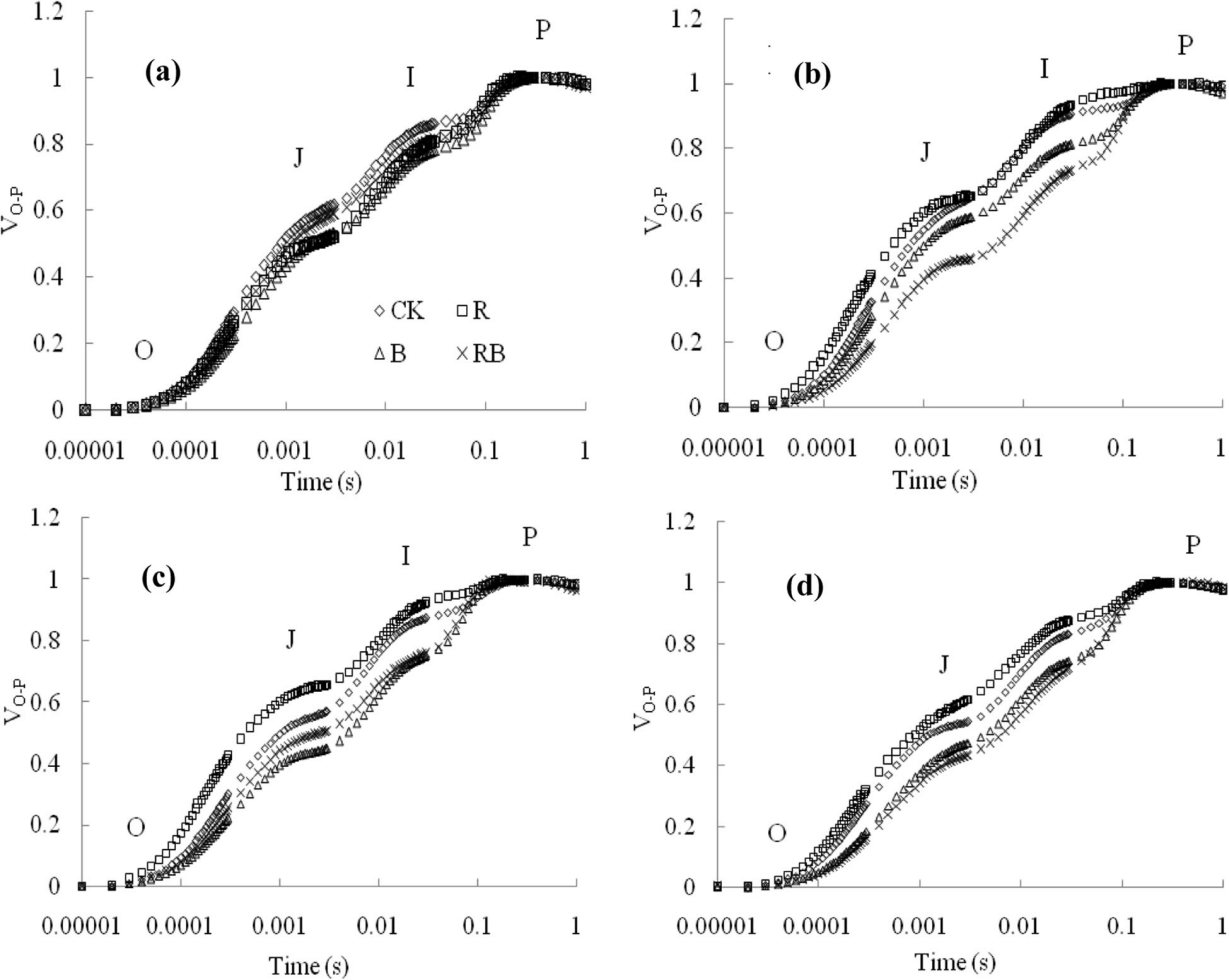
Effects of different light treatments on chlorophyll a fluorescence transient (OJIP) of sweet pepper seedlings at different experimental periods. (**a**), (**b**), (**c**), and (**d**) were at 7, 14, 21, and 28 day after treatment, respectively. W, white light; R, monochromatic R light; B, monochromatic B light; RB, mixed R and B light of 3:1

**Fig. 7:**
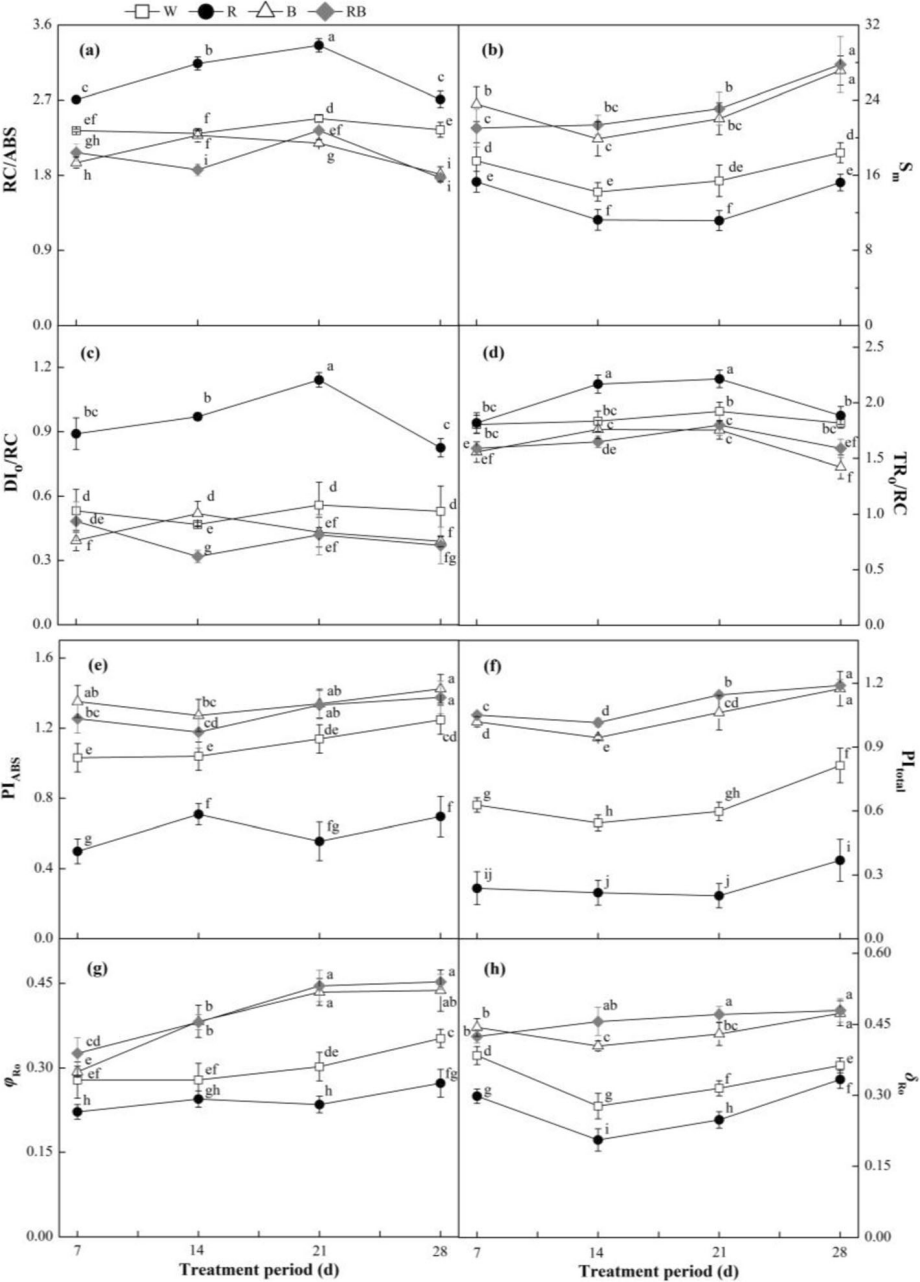
Effects of different light treatments on JIP-test parameters: (**a**) RC/ABS, fraction of PSII Chl a molecules that function as reaction centers; (**b**) S_m_, general electronic carrier of the reaction center; (**c**) DI_o_/RC, dissipated energy in the reaction center; (**d**) TR_o_/RC, maximum trapped energy exciton per active PSII reaction center; (**e**) PI_ABS_, potential for energy conservation from photons absorbed by PSII to the reduction of the intersystem electron acceptors; (**f**) PI_total_, potential for energy conservation from photons absorbed by PSII to the reduction of PSI end acceptors; (**g**) *Φ*_Ro_, quantum yield for reduction of end electron acceptors at the PSI acceptor side; (**h**) *δ*_Ro_, efficiency/probability with which an electron from the intersystem electron carriers is transferred to reduce end electron acceptors at the PSI acceptor side of sweet pepper seedlings at different experimental periods. Data are presented as means ± SE, n = 3. Different letters indicate significant differences between values (*p* < 0.05). W, white light; R, monochromatic R light; B, monochromatic B light; RB, mixed R and B light of 3:1. □ W; ● R; △ B; ◆ RB

The typical polyphasic Chl a fluorescence transient (OJIP) increased at different experimental time points were shown in Fig. 6a-d. In general, the results indicated that the W, B and RB treatments decreased the amplitude of the OJIP curves compared with R, mainly at the J and I step, whereas they were higher under R light. There was no obvious difference in the maximal amplitude of the O and P steps among the treatments (*P* > 0.05). In order to further study the mechanisms behind the observed changes, the JIP-test was used for the fluorescence induction transients (Fig. 7a-h). Most JIP-test parameters (e.g., the general electron carrier of the reaction center (S_m_), the potential for energy conservation from photons absorbed by PSII to the reduction of the intersystem electron acceptors (PI_ABS_), the potential for energy conservation from photons absorbed by PSII to the reduction of PSI end acceptors (PI_total_), the quantum yield for reduction of end electron acceptors at the PSI acceptor side (*Φ*_Ro_) and the efficiency/probability with which an electron from the intersystem electron carriers is transferred to reduce end electron acceptors at the PSI acceptor side (*δ*_Ro_)) were significantly elevated by B and RB compared with W (*P* < 0.05), but the R light produced relatively lower values. Additionally, the fraction of PSII Chl *a* molecules that function as reaction centers (RC/ABS), the dissipated energy in the reaction center (DI_o_/RC) and the maximum trapped energy exciton per active PSII reaction center (TR_o_/RC) in the leaves under R were significantly greater than those under other treatments (*P* < 0.05).

### Calvin cycle enzymes activity under different light treatments

**Fig. 8:**
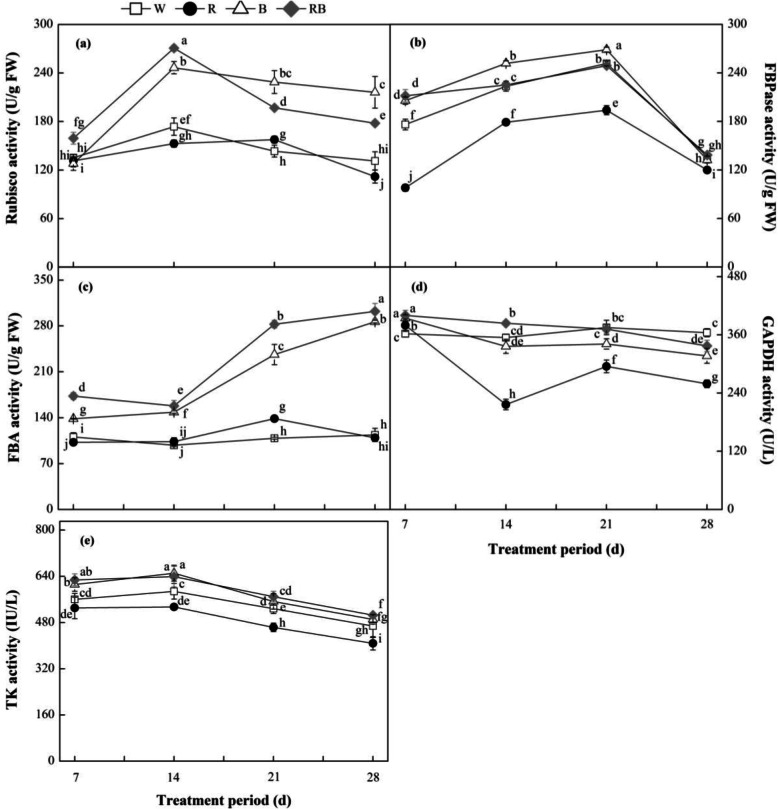
Effects of different light treatments on activities of Calvin cycle-related enzymes: (**a**) Rubisco, ribulose-1, 5-bisphosphate carboxylase/oxygenase; (**b**) FBPase, fructose-1, 6-bisphosphatase; (**c**) FBA, fructose-1, 6-bisphosphate aldolase; (**d**) GAPDH, glyceraldehyde-phosphate dehydrogenase; (**e**) TK, transketolase from sweet pepper seedlings at different experimental periods. Data are presented as means ± SE, n = 3. Different letters indicate significant differences between values (*p* < 0.05). FW, fresh weight; W, white light; R, monochromatic R light; B, monochromatic B light; RB, mixed R and B light of 3:1. □ W; ● R; △ B; ◆ RB

Rubisco, FBPase, fructose-1, 6-bisphosphate aldolase (FBA), glyceraldehyde-phosphate dehydrogenase (GAPDH) and transketolase (TK) are key enzymes in the Calvin cycle. The results showed that the Rubisco activities increased initially and then decreased with the duration of different light quality treatments increased (Fig. 8a-e). Seedlings under B and RB had significantly higher Rubisco activities than W-grown seedlings (*P* < 0.05) with 65 and 36% increases, respectively, at 28 DAT (Fig. 8). In contrast, R-grown plants had a significantly lower activity levels (15% less) than W-grown plants.

Sharp increases in FBPase activity were observed in pepper seedlings under the different light treatments. The FBPase activities reached their highest levels at 21 DAT and then decreased over the following days (Fig. 8b). Activities of this enzyme in plants under B light remained significantly higher than those under other treatments from 7 to 21 DAT (*P* < 0.05), but there was no significant difference between W and B at 28 DAT (*P* > 0.05). Significantly lower activities were observed under R light than those under other treatments during the experimental period. The FBA activities in plants treated with W and R light increased slowly during the experimental period (Fig. 8c), whereas, they rapidly increased in the RB and B treatments after 14 DAT, which indicated that the enzyme activity in the RB and B treatments was greater than in the W and R treatments. The GAPDH activities decreased in plants under all treatments, but the W and RB light applications alleviated the reduction (Fig. 8d). The TK activities were similar under all the treatments during the experimental period, except that the GAPDH and TK activities were significantly lower under the R-treatment than those under other treatments (Fig. 8e).

### Gene expression under different light treatments

**Fig. 9:**
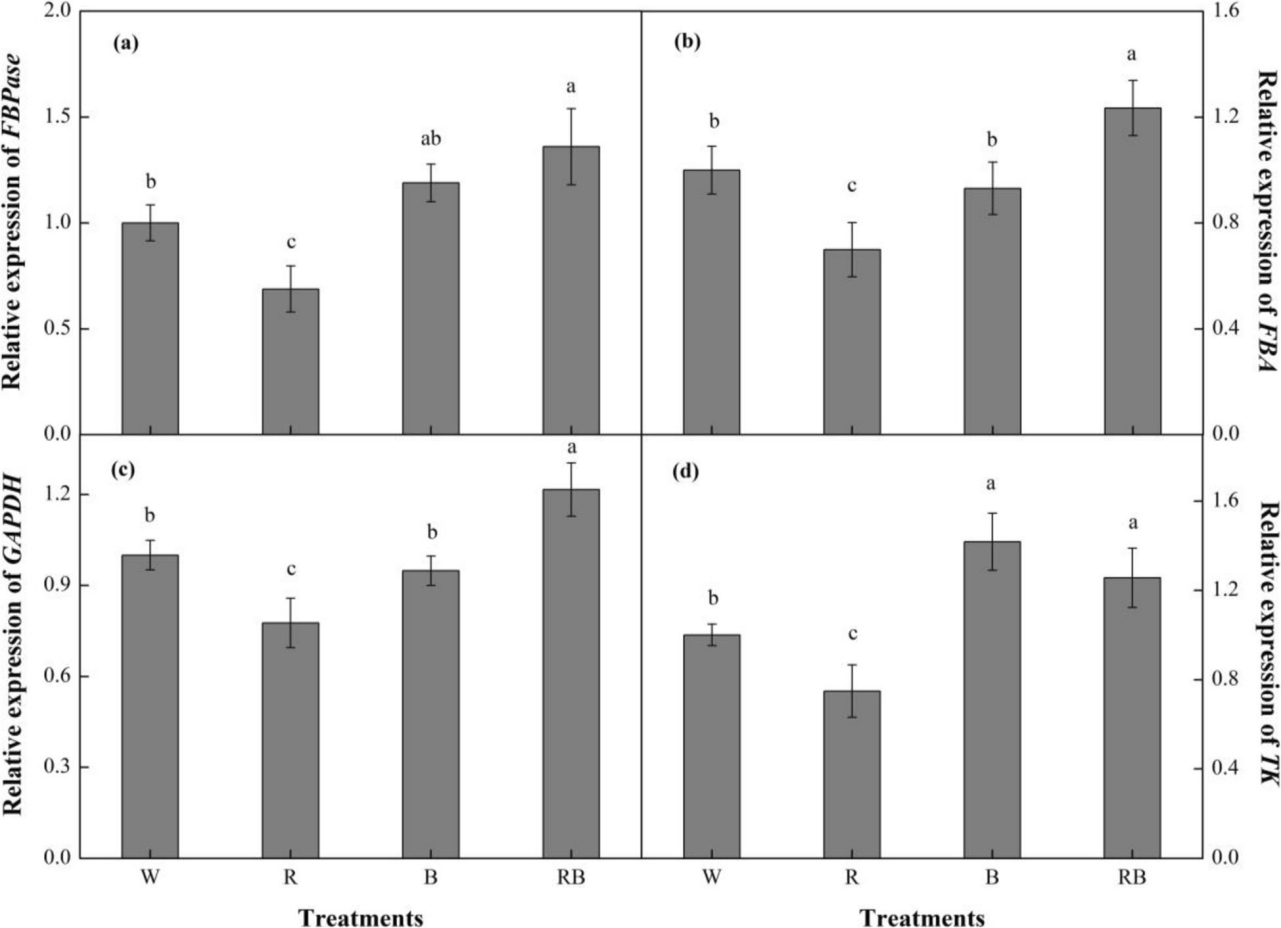
Effects of different light treatments on expression of (**a**) *FBA*; (**b**) *FBPase*; (**c**) *GAPDH*; (**d**) *TK* from sweet pepper seedlings at 28 day after treatment. Data are presented as means ± SE, n = 3. Different letters indicate significant differences between values (*p* < 0.05). W, white light; R, monochromatic R light; B, monochromatic B light; RB, mixed R and B light of 3:1

The RT-PCR method was used to analyze the relative expression levels of *FBA*, *FBPase*, *GAPDH* and *TK* genes involved in the Calvin cycle after pepper seedling exposure to different light qualities for 28 d. Figure 9a-d showed that the transcriptional levels of these genes varied significantly depending on the light qualities supplied and similar variation patterns were obtained for *FBA*, *FBPase* and *GAPDH* under different treatments. Generally, compared to W, seedlings under RB showed significantly increased expression levels of these three genes, whereas exposure to R light resulted in decreased gene transcription. Additionally, the relative expression level of *TK* was up-regulated in B-treated seedlings, followed by RB and W, but R produced the lowest *TK* levels.

## Discussion

During the process of light-controlled growth, it is stated that photoreceptors modulate light-responsive nuclear genes by perceiving and interpreting incident light and transduce signals. In the light spectra, R and B wavelengths can strongly affect plant photosynthesis, physiological metabolism and morphology as the main spectral wavelengths [37–39]. In this study, the photomorphogenesis and photosynthetic characteristics of sweet pepper seedlings were significantly influenced by the light qualities. Biomass is an important indicator of seedling quality. In this study, the seedling DW under RB was significantly greater than those under other treatments, which suggested that this spectrum was optimal because it promoted plant development and drove photosynthesis by increasing Chl *a* and total Chl contents in the seedlings [33, 40]. Previous studies also found that mixed R and B light could promote fresh weight (FW) and DW in many other plant species, such as chrysanthemum, upland cotton and tomato [41–43]. The biomass of pepper seedling was significantly increased under RB compared with other treatments and this was probably due to the enlarged leaf area (LA) [44] and changes to the leaf anatomy.

Light is absorbed by chloroplasts when it passes through the PT and SPT, which are both important photosynthetic tissues. In our study, RB treatment greatly increased the PT, SPT, as well as upper and lower epidermis thickness, which led to thicker leaves, and this was consistent with the results of Arena et al. [45] and Liu et al. [46]. The vertically elongated PT cells minimized light scattering, which allowed deeper penetration into the chloroplasts, while the changes to the SPT cells enhanced light capture by scattering the light [47]. This improved the photosynthetic structure, which should increase the light capture and absorbance capacities, and contribute to better photosynthetic light acclimation. In addition, leaf thickness plays a key role in determining space availability for chloroplast development [48]. The RB treatment increased leaf thickness, which enhanced the chloroplast ultrastructure [49]. The results suggested that a larger LA and increased leaf, as well as PT and SPT cells thickness improved light interception by the pepper seedlings. and this could be another important reason why RB was able to improve photosynthetic efficiency. Furthermore, the thinner leaves recorded under R light can be explained as a reaction to radiation stress on plant development and metabolic processes, as suggested by Macedo et al. [50].

The ability to do well out of the increments in optical energy and CO_2_ of plants is reflected by the light- and CO_2_-response curves, which provides interesting opinions on the mechanisms based on light capture and CO_2_ fixation. In this study, Pn-PPFD under the different light qualities was significantly lower than Pn-CO_2_. This might be due to a CO_2_ concentration limitation. The AQY and CE values showed the initial slopes of the light- and CO_2_-response curves, respectively. They stand for the ability to obtain low levels of light energy and CO_2_ of plants. Our results confirmed a previous study [51], which showed that mixed R and B light promoted AQY and CE, and that these increases led to a rise in Pn_max_ and maximized the RuBP regeneration rate. The RB light led to significant increases in AQY, CE, Pn_max_ and the maximum RuBP regeneration rate. This indicates that mixed R and B light exerts an synergistic effect on increasing photosynthetic capacity [52]. The LSP values, which reflect the plant ability to use the highest light intensity level, were also significantly higher under RB. This showed that RB improved the ability of the leaves to utilize mixed light qualities. Furthermore, the LCP and CCP values were significantly decreased under RB, which showed that this treatment improved photosynthetic performance and light energy utilization efficiency. These results indicated that the energy conversion of mixed R and B light into chemical energy by the leaves was very efficient, as this fraction of visible light had, by far, the highest quantum yield for CO_2_ fixation compared with other light treatments [53].

Light qualities can regulate photosynthesis by affecting the formation of different types of chloroplast proteins and electron transport between light systems [54]. Chl fluorescence can partly reflect the photosynthetic ability of plants [55] and the efficiency of PSII photochemistry (*Φ*_PSII_) can be used to reveal the physiological state of plants [56]. Our results showed that there was a reduction in *Φ*_PSII_ in pepper seedlings after exposure to the RB treatment. *F*_v_/*F*_m_ represents the maximal efficiency of the excitation energy captured by the PSII reaction centers and the significantly higher value observed in RB-treated seedlings indicated that resistance to photoinhibition was up-regulated under this treatment [57]. Additionally, the higher *F*’_v_/*F*’_m_ and *Φ*_PSII_ levels under RB treatment showed that mixed R and B light increased the openness and electron transport efficiency of PSII, which meant that more electrons could be absorbed, captured and transported.

There is a correlation relationship between the J-step, I-step and IP phases of Chl fluorescence transients and the redox states of quinone electron acceptor (Q_A_), plastoquinone and the end acceptors at the side of PSI electron acceptor [58, 59]. The finding that R-treated leaves increased the J- and I-step suggested that electron transport at both the donor and acceptor sides of PSII was inhibited. Therefore, CO_2_ assimilation was decreased by the imbalance of excitation energy distribution between PSI and PSII. Monochromatic B and mixed R and B light induced a decrease in all the OJIP steps during the experimental period compared with other treatments, which altered both the donor and acceptor sides of PSII and affected electron transport [60]. These changes maintained electron transportation on both the donor and acceptor sides. Furthermore, we found that RB increased S_m_, PI_ABS_, PI_total_, *Φ*_Ro_ and *δ*_Ro_, but decreased RC/ABS, DI_o_/RC and TR_o_/RC (Fig. 7), which less damaged the photochemical and non-photochemical redox reactions, enhanced the ability of electron transport and sped up ATP synthesis and RuBP regeneration [61].

In C3 plants, the Calvin cycle is the predominant pathway for CO_2_ assimilation [62]. Rubisco is a representative and unique enzyme in the Calvin cycle and other Calvin cycle enzymes, including FBPase, FBA, GADPH and TK, play an important part in modulating this pathway [63, 64]. As a significant environmental signal, light provokes gene expression and regulates related enzyme activities during the growth of plants. How light adjusts the expressions and activities of enzymes in photosynthesis was examined by several researches [52, 65]. These previous studies were verified by the present study. The Rubisco activity in B- and RB-treated plants was significantly higher than those in the plants treated with other light wavelengths. This finding suggested that the application of B or RB could increase carbon assimilation and RuBP regeneration in the Calvin cycle. It was also found that under R light, photosynthetic rate has decreased as the number of Rubisco activities and the transcriptional levels of most genes in the Calvin cycle reduced. This result was consistent with an earlier observation and implied that the inhibition of CO_2_ carboxylation in the Calvin cycle and PSII slow down as a result of the impaired activity of Rubisco activase, which removes inhibitors bound to Rubisco, are probably responsible for the decreased CO_2_ assimilation rate in R-grown seedlings compared with other light treatments [36, 66]. Furthermore, according to a previous research, the stomatal factor regulating the availability of RuBP differentially, and CO_2_ may participate in adjusting gene expression because there is a high correlation between the expression levels of the genes examined and the changes in stomatal conductance [36].

The FBA and FBPase activities directly affect photosynthetic efficiency and carbon accumulation [67]. Furthermore, a previous study showed that a significantly decrease in TK activity led to a significant reduction in RuBP regeneration and significantly inhibited the plant photosynthetic rate [68]. In our study, the activities of these enzymes under B and RB and the relative expression of their associated genes, except for *FBA* and *TK*, were significantly elevated, which promoted RuBP regeneration and increased Pn [67, 68]. Chloroplast GAPDH is a key enzyme involved in the carbon reduction process during photosynthesis [69] and the greater *GAPDH* expression level under RB light in the present study may be due to the increased demand for carbon flux [70], suggesting that maintenance of active *GAPDH* expression in the carbon reduction process could be an important factor contributing to superior photosynthesis under RB light [71]. Changes in activities of FBA and TK as well as their expression under all treatments were not positively correlated, suggesting that transcript abundance is poorly linked to de novo protein synthesis due to profound regulation at the level of translation Oelze et al. [72]. Moreover, the different patterns of gene expression and activity are probably correlated with regulatory factors other than light quality, but this needs further investigation.

## Conclusions

Light quality is an important environmental factor that regulates the plant photomorphogenesis and photosynthetic characteristics. In conclusion, sweet pepper growth, development and photosynthesis are precisely controlled and genetically regulated by light quality. The results indicated that photosynthesis in seedlings under R light was inhibited by the decreased photosynthetic electron transport capacity, which caused a reduction in CO_2_ assimilation. This led to down-regulation of Calvin cycle associated gene expressions and their related enzymatic activities. However, the use of monochromatic B and mixed R and B light, especially the latter, could enhance the activity of the PSII reaction center and improve photosynthesis and the expression and activities of Calvin cycle-related enzymes, including Rubisco, FBPase and GAPDH, which are probably the main enzymatic factors contributing to RuBP synthesis. Therefore, mixed R and B light may provide more suitable light conditions for the growth of sweet pepper seedlings.

## Methods

### Plant material and climate conditions

The experiment was performed from June to October, 2016 in a Chinese solar greenhouse (CSG) and an artificial climate chamber (ACC, Zhejiang Qiushi Environment Co., Zhejiang, China) at the Horticultural Research Center, Shandong Agricultural University, P. R. China. After immersing sweet pepper (*Capsicum annuum* L. cv. Hongqijian) seeds (Jinan Weili Seeds Co., Ltd., Shandong, China) in water for 15 min at the temperature of 55 °C and soaking it in cold water (4 °C) for 24 h. The seeds were sown into 50-cell plug trays (54.0 × 30.0 × 4.4 cm) filled with a mixture of peat (Floragard Seed 2, Floragard Co., Oldenburg, Germany) and vermiculite (2:1, v/v) in the CSG. All seedlings were watered daily with half-strength Yamazaki’s pepper nutrient solution. Three weeks later, when their second true leaf had fully expanded, the seedlings were transplanted into plastic pots (8 cm long, 8 cm wide and 10 cm deep, one seedling per pot) containing the same substrate and watered with full-strength nutrient solution. Then, 480 seedlings in total were chosen, transferred into the ACC and cultured while receiving four kinds of light quality treatments for 28 d. Each light treatment was repeated three times in the same ACC and there were 40 plants for per replication per treatment. Five plants were randomly sampled at 7, 14, 21 and 28 DAT from each replication each treatment and were subjected to morphological and biochemical analyses. There was ventilation in the controlled environment, so the CO_2_ level was the same as the CO_2_ level of atmosphere outside. The relative humidity (RH) was kept at 70 ± 10%, with a 12 h photoperiod and a temperature of 26 ± 1 °C during the daytime and 18 ± 1 °C at night.

### Light treatments

All the mixed LEDs had a uniform spectrum for R and B light and were designed by Chunying Optoelectronics Technology Co., Ltd., Guangdong, China. The cultivation rack in the ACC was a steel frame structure with an LED light source placed at the top. The different treatments were insulated from one another by silver shading material. The plants were grown under the following light conditions: monochromatic B light with a maximum intensity at 457 nm, R light or mixed R and B light (3:1, RB: 75% R light witht a wavelength of 657 nm and 25% B light with a wavelength of 457 nm) has a maximum intensity at 657 nm. There was a multi-wavelength W light treatment as control (Supplementary Fig. 1). The light intensity, expressed as PPFD at the canopy level, was set at 300 μmol/m^2^·s, which was measured using a quantum sensor (LI-250, LI-COR Inc., Lincoln, NE, USA) and maintained by adjusting the distance of the LEDs from the canopies. The LEDs was approximately 10 cm far away from the canopy. A spectroradiometer (Unispec-SC Spectral Analysis System, PP Systems Inc., Haverhill, MA, USA) was used to measure the spectral photon flux density distributions (SPDs) of the LEDs.

### Biomass analysis

Five seedlings, including leaves and roots, were removed from each replication each treatment at 28 DAT and dried in an oven at 105 °C for 30 min. The oven temperature was changed to 75 °C and the plants were dried to a constant weight. Then, the DWs of leaves and roots were measured using an electronic balance (precision: ± 0.1 g, Model LA16001S, Sartorius Co., Hamburg, Germany).

### Leaf anatomy

Leaf anatomy was measured on the fully expanded second leaves from five pepper seedlings at a similar position for each replication each treatment [46] on 28 DAT. Leaf segments of 5 mm × 5 mm were taken from the central leaf blade next to the main vein, fixed with formalin-acetic acid-alcohol (FAA) fixative, dehydrated in an alcohol and xylene series, embedded in paraffin, cross-sectioned to a thickness of 10 μm, and stained with red-solid green. The total thickness of the whole leaf and the thickness of the upper epidermis, lower epidermis, PT and SPT were measured under a transmission light microscope (DP71, Olympus Inc., Tokyo, Japan). Images were collected using a digital camera (Camedia C4040, Olympus Inc., Tokyo, Japan) and analyzed by AnalySIS 5.0 (Olympus Inc., Tokyo, Japan).

### Photosynthetic light- and CO_2_-response curves

Between 09:00 am and 14:00 pm, the measurement of photosynthetic light-response curves and CO_2_-response curves was made on the second leaf fully-unfolded using a portable photosynthesis systems machine (LI-6400XT, Li-COR, Lincoln, NE, USA) at 28 DAT. The measurement technique was based on a modified method described by Pan et al. [52]. In the leaf chambers, the temperature was 26 ± 1 ∘C, air relative humidity was 65 ± 5% and the flow rate was 300 μmol/s. The measurement of light-response curves was made under different graded PPFD series of 1800, 1500, 1200, 1000, 800, 600, 400, 300, 200, 150, 100, 50, 20 and 0 μmol/m^2^·s. When the CO_2_-response curve measurements were taken, the light intensity and CO_2_ concentration of the leaf cuvette were set to 1000 μmol/m^2^·s and 400 μmol/mol, respectively, for 30 min. After reaching a steady state, the curves of CO_2_ response were measured by a CO_2_ mixer under a graded Ci value series of 400, 300, 200, 100, 50, 100, 200, 300, 400, 600, 800, 1000, 1200, 1500 and 1800 μmol·CO_2_/mol. The leaf chamber spends 120 to 180 s in adjusting its new microclimate each time. According to a previous report, three times of measurement were made for each curve, which was suitable for a non-linear regression equation [73, 74], so that the LCP, LSP, Pn_max_, CCP, CSP and the maximum RuBP regeneration rate. The starting slope of the curve of light response was the AQY, and the starting slope of the curve of CO_2_ response was the CE.

### Chlorophyll fluorescence and chlorophyll fluorescence transients

The Chl fluorescence measurements were performed using a portable pulse modulation fluorometer (FMS-II, Hansatech Instruments Ltd., King’s Lynn, Norfolk, UK). The second fully expanded leaves of five seedlings from each replication each treatment were dark adapted for 20 min, and the *F*_o_ (original fluorescence yield) and *F*_m_ (maximum fluorescence yield) were determined. Then, the leaves were put under natural light for 1 h, and the measurements of *F*’_o_, *F*’_m_ and *F*_s_ values was made under the activating light of 800 μmol/m^2^·s. With the saturation pulse intensity of 3000 μmol/m^2^·s and the duration of 0.8 s, *F*’_o_ and *F*’_m_ respectively stand for the minimum and maximum fluorescence yields of an illuminated leaf, which were measured by applying the method of saturation pulse. *F*_s_ means the steady fluorescence yield. The maximum photochemical efficiency of PSII was calculated using *F*_v_/*F*_m_ = (*F*_m_ – *F*_o_) / *F*_m_, actual PSII photochemical efficiency was calculated using (*Φ*_PSII_) = (*F*’_m_ – *F*_s_) / *F*’_m_ and maximum photochemical efficiency of PSII under light adaptation was calculated using (*F*’_v_/*F*’_m_) = (*F*’_m_ – *F*’_o_) / *F*’_m_.

A plant efficiency analyzer (Handy PEA, Hansatech Instruments Ltd., King’s Lynn, Norfolk, UK) was used to measure the OJIP on the second leaves. Strasser’s method was employed to calculate the JIP-test formulae and glossary of terms [75, 76]. The following derivative parameters were determined according to Lin et al. [61] and Miao et al. [30]: RC/ABS, S_m_, DI_o_/RC, TR_o_/RC, PI_ABS_, PI_total_, *Φ*_Ro_ and *δ*_Ro_.

### Calvin cycle enzymes activity

After being sampled at 7, 14, 21 and 28 DAT, the second leaves selected from top 15 plants of each treatment were used to determine the enzyme activities. Leaf tissue (0.5 g) was homogenized in 4 mL of ice-cold extraction buffer: (25 mM Hepes (K^+^), pH 7.5, 10 mM MgSO_4_, 5 mM dithiothreitol (DTT), 1 mM Na_2_EDTA, 1 mM phenylmethanesulfonyl fluoride (PMSF), 5% (w/v) insoluble polyvinylpyrrolidone (PVP) and 0.05% (v/v) Triton X-100). The homogenate was filtered through muslin cloth and centrifuged at 14,000×g for 5 min at 4 °C. The supernatant was used as the enzyme extract for the enzyme activity assays [77].

An ELISA kit (Shanghai Yanji Biological Technology Ltd., Shanghai, China) was employed to determine the Rubisco (EC 4.1.1.39), FBPase (EC 3.13.11), FBA (EC 4.1.2.13), GAPDH (EC 1.2.1.12) and TK (EC 2.2.1.1) activities, and the extraction approach for these enzymes were modified based on Rao and Terry [78] and Wang et al. [36]. After grounding the frozen leaf samples (0.5 g) to fine powder in a liquid nitrogen with a mortar and pestle, the powder was put into a centrifuge tube and extracted to the precool extraction buffer (5 mL). The centrifugation of enzyme extraction solution was made at 12,000×g for 15 min at the temperature of 4 °C. The activity assay of Calvin cycle enzymes used the supernatant. Afterwards, a microplate absorbance reader (Bio-Tek ELX800, Bio-Tek Instruments, Winooski, VT, USA) was used to determine the activities of the Calvin cycle enzymes under an absorbance of 450 nm based on the instructions of the manufacturer.

The measurement of the protein concentration of each enzyme extraction solution was made based on Bradford [79]. The results of the measurement were showed as U/g of protein.

### Gene expression

Quick RNA Isolation Kit was used to extract total RNA according to the supplier’s instructions (Huayueyang Biotech Co., Ltd., Beijing, China). A ReverTra Ace qPCR RT-Kit (Toyobo Bio-Technology, Co., Ltd., Osaka, Japan) was applied to make reverse transcription. Real-time PCR was employed to conduct the gene expression analysis with 18S rRNA as an internal control. The thermal cycler procedure was cycled once for 2 min at the temperature of 94 °C and cycled for 40 times at the temperature of 94 °C for 10 s, 60 °C for 20 s and 72 °C for 30 s. The method described in Livak and Schmittgen was used to analyze relative gene expressions [80]. The specific gene primers used for real-time PCR analysis of the genes involved in the PS complexes are shown in Supplementary Table 1.

### Data analysis

The experiment had a totally random design. Values presented are the mean ± standard deviation (SD) of three replicates. One-way variance analysis (ANOVA) was employed to analyze the data, and the differences between the means were tested by Duncan’s multiple range test (*P* < 0.05). The charts were created using Origin (version 8.5, Microcal Software Inc., Northampton, MA, USA).

## Supplementary information

**Additional file 1.**

## Data Availability

All data generated or analyzed during this study are included in this published article and its supplementary information files.

## Abbreviations

R: Red
B: Blue
W: White
RB: Mixed red and blue light
PPFD: Photosynthetic photon flux density
Chl: Chlorophyll
ETR: Electron transport rate
NPQ: Non-photochemical quenching
LED: Light-emitting diode
LA: Leaf area
CSG: Chinese solar greenhouse
ACC: Artificial climate chamber
D: Day
DAT: Day after treatment
RH: Relative humidity
SPD: Spectral photon flux density distribution
DW: Dry weight
FAA: Formalin-acetic acid-alcohol
EP: Epidermis cell
PT: Palisade mesophyll tissue
SPT: Spongy mesophyll tissue
Pn: Net photosynthetic rate
LCP: Light compensation point
LSP: Light saturation point
Pn_max_: Light-saturated maximum
CCP: CO_2_ compensation point
CSP: CO_2_ saturation point
AQY: Apparent quantum efficiency
CE: Carboxylation efficiency
*F*_v_/*F*_m_: Maximum photochemical efficiency of PSII
*Φ*_PSII_: Actual PSII photochemical efficiency
*F*’_v_/*F*’_m_: Maximum photochemical efficiency of PSII under light adaptation
OJIP: Chl *a* fluorescence transient
RC/ABS: Fraction of PSII Chl *a* molecules that function as reaction centers
S_m_: General electronic carrier of the reaction center
DI_o_/RC: Dissipated energy in the reaction center
TR_o_/RC: Maximum trapped energy exciton per active PSII reaction center
PI_ABS_: Potential for energy conservation from photons absorbed by PSII to the reduction of the intersystem electron acceptors
PI_total_: Potential for energy conservation from photons absorbed by PSII to the reduction of PSI end acceptors
*Φ*_Ro_: Quantum yield for reduction of end electron acceptors at the PSI acceptor side
*δ*_Ro_: Efficiency/probability with which an electron from the intersystem electron carriers is transferred to reduce end electron acceptors at the PSI acceptor side
RuBP: Ribulose-1, 5-bisphosphate
Rubisco: Ribulose-1, 5-bisphosphate carboxylase/oxygenase
FBPase: Fructose-1, 6-bisphosphatase
FBA: Fructose-1, 6-bisphosphate aldolase
GAPDH: Glyceraldehyde-phosphate dehydrogenase
TK: Transketolase
DTT: Dithiothreitol
PMSF: Phenylmethanesulfonyl fluoride
PVP: Polyvinylpyrrolidone
