# Supplementary material for: Effects of red and blue light on leaf anatomy, CO2 assimilation and the photosynthetic electron transport capacity of sweet pepper (Capsicum annuum L.) seedlings
Source: BMC Plant Biol. 2020 Jul 6;20:318. doi: 10.1186/s12870-020-02523-z (PMC7336438; doi:10.1186/s12870-020-02523-z)
Supplement: Supplementary file 1 — Additional file 1. [file 12870_2020_2523_MOESM1_ESM.docx]

**Table 1** Primes sequences used for real time RT-PCR assays.

| Gene | Forward primer | Reverse primer |
| --- | --- | --- |
| *actin* | 5'-TGAAAATCAAGGTGGTGGCG-3' | 5'-TCCGGTGAACAATGGAAGGA-3' |
| *FBA* | 5'-ATGAACCAAGCACCAAACCC-3' | 5'-TGTACTTTCCGAGCTGAGCA-3' |
| *FBPase* | 5'-TCGTCAGCCATTTCTTCCCT-3' | 5'-CACCCACATCAACTCCTCCT-3' |
| *GAPDH* | 5'-GACTACTGTGCACGCAACAA-3' | 5'-AGCAGCACCAGTTGAACTTG-3' |
| *TK* | 5'-CCCAATGTTCTGATGCTCCG-3' | 5'-CCACCCTTTGCTGTTCCTTC-3' |


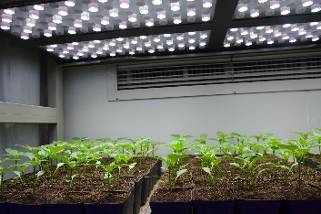

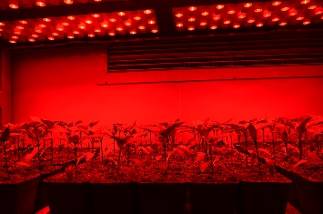

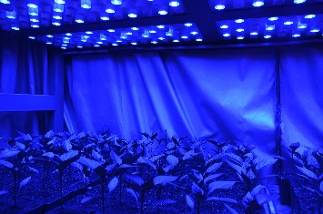

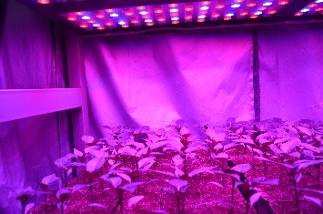

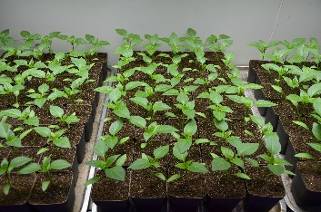

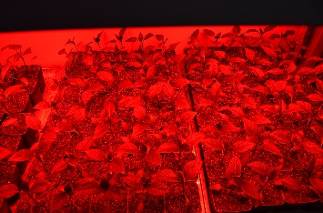

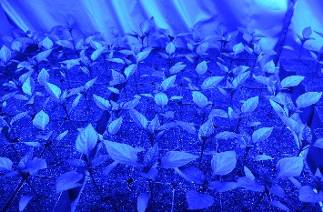

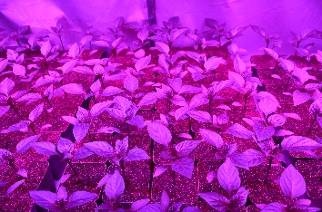


**W R B RB**


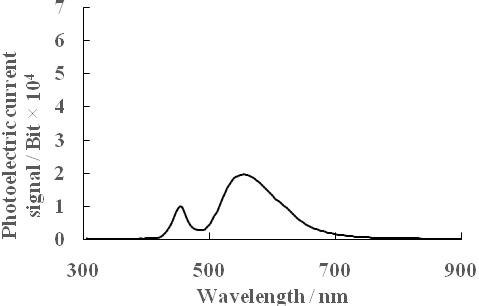

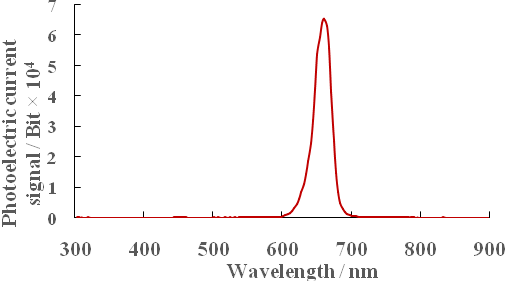

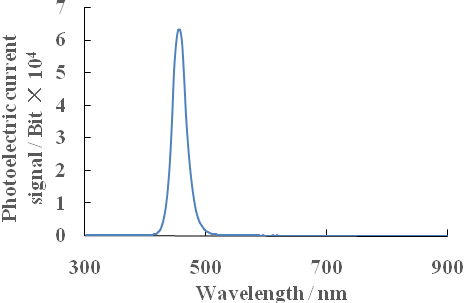

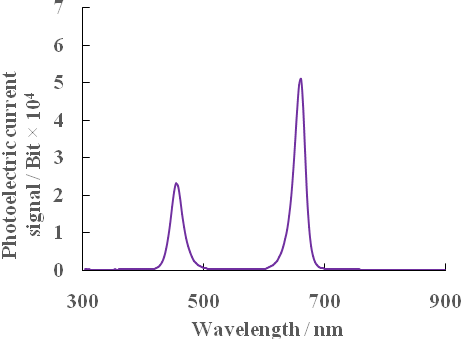


**(a)**

**(b)**

**Fig. 1.** (a) Characteristics of the respective LED irradiance spectra in the different treatments (upper photographs); (b) General view and spectral distribution of light quality treatments (lower photographs). W, white light; R, monochromatic R light; B, monochromatic B light; RB, mixed R and B light of 3:1.


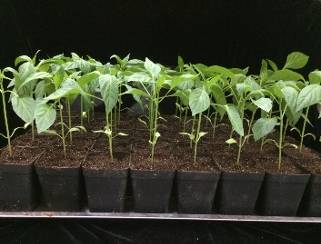

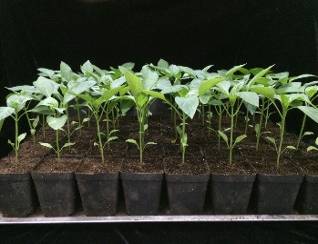

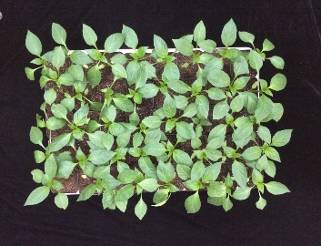

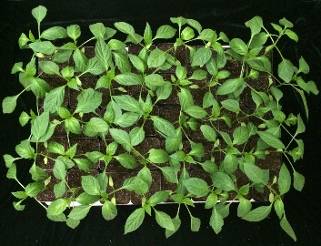

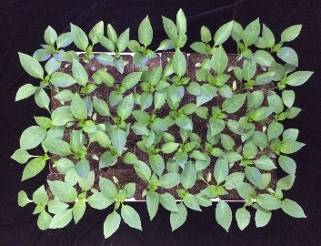

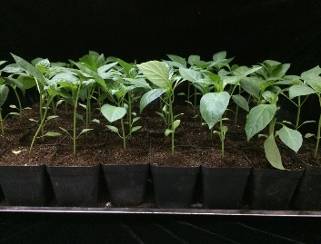

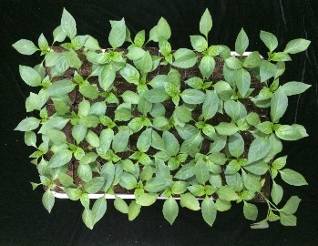

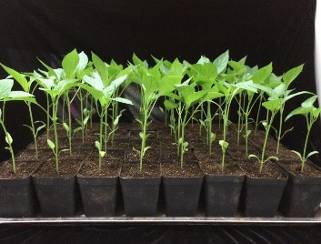


**W R B RB**

**Fig. 2.** Side (upper photographs) and top (lower photographs) views of sweet pepper seedlings at 28 day after treatment under different light treatments. W, white light; R, monochromatic R light; B, monochromatic B light; RB, mixed R and B light of 3:1.
